# Comparison of the dietary fibre composition of old and modern durum wheat (*Triticum turgidum* spp. *durum*) genotypes

**DOI:** 10.1016/j.foodchem.2017.09.143

**Published:** 2018-04-01

**Authors:** Michele Andrea De Santis, Ondrej Kosik, Diana Passmore, Zina Flagella, Peter R. Shewry, Alison Lovegrove

**Affiliations:** a Dipartimento di Scienze Agrarie, degli Alimenti e dell’Ambiente (SAFE), Università degli Studi di Foggia, Via Napoli 25-71122, Foggia, Italy; b Rothamsted Research, Harpenden, Hertfordshire AL5 2JQ, United Kingdom

**Keywords:** AX, arabinoxylan, AXOS, arabinoxylan oligosaccharides, GPC, grain protein content, GOS, β-glucan oligosaccharides, MLG, mixed-linkage-β-glucan, SPC, semolina protein content, RV, relative viscosity, TKW, thousand kernel weight, TW, test weight, YR, year of release, Arabinoxylan, β-Glucan, Dietary fibre, Durum wheat, Old and modern genotypes, Viscosity

## Abstract

It has been suggested that intensive breeding has led to decreased contents of health-promoting components in modern wheats. We therefore compared the contents and compositions of the major dietary fibre components, arabinoxylan and β-glucan, in semolina and wholemeal flour of old and modern Italian durum wheats. No differences in total arabinoxylan content were observed but the modern varieties had higher proportions of soluble arabinoxylan in wholemeals and of β-glucan in semolina. The study therefore provides no evidence that intensive breeding has had negative effects on the contents of dietary fibre components in durum wheat. However, comparison of material grown over two years indicated that the content and composition of arabinoxylan and β-glucan were more stable in the older than in the modern genotypes. The identification of modern cultivars with high viscosity associated with a high content of β-glucan suggests that they are good sources of fibre for human health.

## Highlights

• Dietary fibre components in old and recent genotypes of durum wheat are compared.
• No differences in total arabinoxylan were observed in wholemeal or semolina.
• Recent genotypes contained a higher proportion of soluble arabinoxylan in wholemeal.
• Recent genotypes contained higher proportions of beta-glucan.
• Intensive breeding has not led to decreases in dietary fibre in durum wheat.

## 1 Introduction

Wheat is the most widely cultivated crop in the world and the dominant staple crop in temperate countries, where it provides between 20% and 50% of the total intake of calories. However, most of the wheat grown globally is bread wheat, with durum wheat (pasta) (*Triticum turgidum* var. *durum*) accounting for only about 5–6% of the total wheat production. Durum wheat is predominately cultivated in the Mediterranean basin and in other regions with a similar climate (Shewry & Hey, 2015), with the flour (semolina) being mainly used for making pasta. In addition, durum wheat is also used for making bread, couscous and other traditional foods.

The global success of wheat results from its wide adaptation to local environments and good processing properties, but also from the high yields resulting from intensive breeding carried out over the past century. Such breeding programmes for durum wheat started at the beginning of the 20 th century and were primarily focused on increased grain yield, shorter stature and early maturation to suit Mediterranean conditions where drought and high temperature can occur during ripening (De Vita et al., 2007), but also improved protein quality for pasta making (Raciti, Doust, Lombardo, Boggini, & Pecetti, 2003). However, it has been suggested that the emphasis of intensive breeding on yield and grain quality has resulted in the neglect of components which may contribute to health (such as secondary products which also confer resistance to herbivores) (Morris & Sands, 2006).

Cereals are an important source of dietary fibre (DF), especially in western diets, such as the UK where they account for about 40% of the total DF intake (Bates, Lennox, & Swan, 2011). DF is not digested and absorbed in the small intestine but passes to the colon where it undergoes bacterial degradation (fermentation) (Topping, 2007). Although DF has a range of health benefits, of particular importance is the capacity to lower the glycaemic response of diets, reducing the risk of developing type II diabetes (Nugent, 2005). Soluble fibre is considered to be especially effective in this respect, which may result from increased viscosity which inhibits mixing and diffusion in the intestinal tract and delays gastric emptying (Riccardi et al., 2004).

The principal types of DF are non-starch polysaccharides (NSP), with the major components in wheat grain being arabinoxylan (AX) and mixed-linkage β-glucan (MLG) (Lafiandra et al., 2014, Lovegrove et al., 2015) which represent about 70% and 20%, respectively, of the NSP in wheat starchy endosperm and white flour fractions (Mares & Stone, 1973). AX consists of a backbone of xylose (β-d-xylopyranosyl) linked by (1 → 4) glycosidic linkages, which may be substituted with arabinose (α-l-arabinofuranosyl) at either the 3 or the 2 and 3 positions. AX is present as water-extractable (WE-AX) and water-unextractable (WU-AX) fractions, with average contents of about 0.5 and 1.7 g/100 g flour, respectively (Dervilly-Pinel, Rimsten, Saulnier, Andersson, & Aman, 2001; Ordaz-Ortiz & Saulnier, 2005; Saulnier et al., 1995, Saulnier et al., 2007). MLG consists of glucose units with (1 → 4)-β linkages (as in cellulose), but these are interspersed with (1 → 3)-β-linkages which generally occur after three or four β-(1 → 4) linkages (Li, Cui, & Kakuda, 2006). The irregular linkage structure prevents the formation of an ordered crystalline structure, leading to the MLG being partially water-soluble.

Most previous studies of DF in wheat have focused on bread wheat. For example, the EU HEALTHGRAIN project compared 150 bread wheat and 10 durum wheat cultivars (Ward et al., 2008), showing 1.35–2.75% and 1.70–2.35% total AX (TOT-AX) in white flour, respectively. Analysis of multisite trials showed that 60–70% of the variation in the content of AX (WE and TOT) in white flour was heritable, with about 30% due to the environment and a low effect of genotype × environment interactions (Shewry, Piironen, et al., 2010). However, analysis of the same datasets showed no relationships between the amounts of dietary fibre components and the release dates of bread wheat genotypes (Shewry et al., 2011). More recently, a comparison of 104 tetraploid accessions showed a range 1.5–5.5% of TOT-AX in whole grain flour (Marcotuli et al., 2015). However, although the lines included durum wheats of different years of registration, the relationship of this to composition was not studied.

We have therefore compared the contents and compositions of dietary fibre components in sets of eight modern and seven old Italian durum wheat genotypes, grown in a two-year field trial, to determine whether there has been an impact of breeding during the 20 th century.

## 2 Materials and methods

### 2.1 Plant material

Eight modern and seven older Italian durum wheat (*Triticum turgidum* spp. *durum*) genotypes were selected based on their release dates (from 1900 to 2005) (Table 1). The pedigrees of these genotypes are graphically represented in Supplementary Fig. S1. The older genotypes were released between 1900 and 1949 and comprised four local Italian landraces from Apulia (Dauno III, old Saragolla) and Sicily (Russello, Timilia “*reste bianche*”), Cappelli which was the first Italian durum wheat cultivar obtained from the introduction of north African landraces by Nazareno Strampelli (1866–1942), and Garigliano and Grifoni 235 which were obtained by selection from Cappelli. All of the modern genotypes were released after the introduction of the (Rht) dwarfing genes, dating from 1985 to 2005.

**Table 1:** TOT-AX, WE-AX (determined as pentosan) and AX solubility in semolina and wholemeal and relative viscosity of aqueous extract from semolina of old and modern durum wheat genotypes grown in two years.

Genotype	YR	Semolina								Wholemeal					
		TOT-AX		WE-AX		AX solubility		RV		TOT-AX		WE-AX		AX solubility	
		(g/100 g dm)		(g/100 g dm)		(%)		(ratio)		(g/100 g dm)		(g/100 g dm)		(%)	
		2013	2014	2013	2014	2013	2014	2013	2014	2013	2014	2013	2014	2013	2014
a*Old*															
Dauno III	1900	1.65^a^	1.43^a^	0.47^F–K^	0.51^D–H^	28.8^D–I^	36.9^A–E^	1.49^LM^	1.69^FG^	4.24^A–F^	4.34^A–C^	0.55^E–I^	0.40^KL^	13.0^G–K^	9.2 ^MN^
Old Saragolla	1900	1.80^a^	1.59^a^	0.39^G–M^	0.47^F–J^	22.2^F–I^	30.2^C–H^	1.63^G–I^	1.56 ^J–L^	3.35 ^J^	3.91^B–I^	0.43 ^J–L^	0.51^G–J^	13.0^G–K^	13.6^G–K^
Russello	1910	1.71^a^	1.53^a^	0.49^E–I^	0.51^D–H^	28.7^D–I^	33.4^B–F^	1.57^H–K^	1.62^H–J^	3.75^E–J^	4.27^A–E^	0.43 ^J–L^	0.52^G–J^	11.4 ^J–N^	12.3^I–M^
Timilia R.B.	1910	1.53^a^	1.61^a^	0.46^F–L^	0.61^A–D^	29.1^C–I^	41.0^A–C^	1.64^GH^	1.84^D^	4.20^A–F^	4.14^A–G^	0.38^L^	0.64^D–F^	9.0 ^N^	15.5^C–G^
Cappelli	1915	1.40^a^	1.72^a^	0.39^G–M^	0.58^C–F^	28.0^E–I^	33.5^B–F^	1.55 ^J–L^	1.78^DE^	4.21^A–F^	3.49^IJ^	0.53^F–J^	0.50^G–K^	12.6^H–L^	14.5^E–J^
Garigliano	1927	1.67^a^	1.77^a^	0.45^F–L^	0.55^D–G^	26.9^E–I^	31.2^C–H^	1.46 ^MN^	1.99^C^	4.08^A–H^	3.95^B–I^	0.46^I–L^	0.44^I–L^	11.1 ^K–N^	11.3 ^K–N^
Grifoni 235	1949	1.80^a^	1.77^a^	0.42^F–M^	0.55^D–G^	23.7^F–I^	31.2^C–H^	1.46 ^MN^	1.56 ^J–L^	4.15^A–G^	4.20^A–F^	0.39^KL^	0.52^G–J^	9.7^LMN^	12.5^H–L^

*Modern*															
Adamello	1985	1.57^a^	1.64^a^	0.45^F–L^	0.75^AB^	28.8^D–I^	46.1^A^	1.59^H–K^	2.05^BC^	4.17^A–G^	4.08^B–I^	0.55^E–I^	0.61^D–G^	13.3^G–K^	15.2^D–I^
Simeto	1988	1.45^a^	1.85^a^	0.31 ^K–M^	0.59^B–F^	21.4^G–I^	32.0^C–G^	1.56 ^J–L^	2.04^BC^	4.13^A–G^	3.79^D–J^	0.58^E–H^	0.55^E–I^	14.0^F–K^	14.5^E–J^
Preco	1995	1.60^a^	1.79^a^	0.51^D–H^	0.72^A–C^	31.9^C–G^	40.0^A–D^	2.01^C^	2.28^A^	3.83^C–J^	3.66^G–J^	0.61^D–G^	0.66^DE^	15.9^C–G^	18.0^CD^
Svevo	1996	1.54^a^	1.76^a^	0.34^I–M^	0.65^A–E^	21.6^G–I^	36.7^A–E^	1.57 ^J–L^	2.30^A^	3.57^H–J^	4.15^A–G^	0.77^BC^	0.70^C^	21.8^AB^	16.8^C–F^
Iride	1996	1.50^a^	1.55^a^	0.30^L–M^	0.37^H–M^	20.2^HI^	24.2^F–I^	1.47 ^MN^	1.74^EF^	4.19^A–F^	4.31^A–D^	1.00^A^	0.81^B^	23.8^A^	18.9^BC^
Claudio	1998	1.70^a^	1.38^a^	0.31 ^J–M^	0.32 ^J–M^	23.2^F–I^	22.7^F–I^	1.52 ^K–M^	1.40 ^N^	4.43^AB^	4.56^A^	0.78^BC^	0.79^BC^	17.6^C–E^	17.4^C–E^
Saragolla	2004	1.48^a^	1.91^a^	0.27 ^M^	0.39^H–M^	18.3^I^	20.2^HI^	1.29^O^	1.31^O^	3.72^F–J^	4.27^A–D^	0.46^I–L^	0.47^H–L^	11.1 ^K–N^	12.3^I–M^
PR22D89	2005	1.57^a^	1.84^a^	0.38^H–M^	0.81^A^	24.4^F–I^	44.0^AB^	1.57^I–K^	2.08^B^	4.16^A–G^	4.54^A^	0.47^H–L^	0.82^B^	11.3 ^K–N^	18.0^CD^

b*Old*	<1949	1.64^a^ ± 0.02		0.47^a^ ± 0.02		30.4^a^ ± 1.2		1.63^b^ ± 0.04		4.02^a^ ± 0.05		0.48^b^ ± 0.02		12.1^b^ ± 0.45	
*Modern*	>1949	1.63^a^ ± 0.02		0.49^a^ ± 0.02		28.5^a^ ± 1.1		1.74^a^ ± 0.04		4.09^a^ ± 0.05		0.66^a^ ± 0.02		16.2^a^ ± 0.42	

a genotype × year interaction. Values of each parameter followed by different letters are significantly different at P ≤ 0.05 (small letters) and at P ≤ 0.001 (capital letters) according to the Tukey’s test.

b Mean of old and modern groups with relative standard error. Different letters are significantly different at P ≤ 0.05 according to Student’s *T*-Test.

Grains were obtained from two field trials (in 2013 and 2014) in a randomized complete block design with three replications on a clay–loam soil at Foggia (Italy, 41° 28′ N, 15° 32′ E and 75 m a.s.l.), as reported previously (De Santis et al., 2017). Briefly, nitrogen and phosphorous were applied at 80 kg ha^−1^ and 70 kg ha^−1^, respectively, according to the standard agronomic practices adopted in Mediterranean areas. The two crop seasons were characterised by different levels of rainfall (54 mm and 153 mm respectively in 2013 and 2014) during the grain development stage.

### 2.2 Grain quality analysis

Thousand kernel weight (TKW) and test weight (TW) were determined. Moisture and ash were determined by NIR using an Infratec 1241 Analyzer (Foss, Hillerod, Denmark). Wholemeal and semolina flours were prepared using a Cyclotec Tecator 1093 sample mill (sieve 1 mm) and a laboratory mill (4 cylinders, sieve 180 µm, Bona), respectively. Samples were subsequently re-milled using a Ball mill (sieve 150 µm). Total N contents of wholemeal and semolina flour were determined using the Dumas method (Dumas, 1931) and multiplied by 5.7 to give protein content.

### 2.3 Determination of total and water-extractable pentosans (AX)

Total and water extractable pentosans (AX) were determined using a colorimetric method (Douglas, 1981, Finnie et al., 2006). Briefly, 125 mg of sample were suspended in 25 ml of water, vortexed and 1 ml aliquots, in duplicate, were transferred to new Pyrex tubes (fraction 1, total AX). The remainder of the suspended sample was mixed on a Spiramix tube roller for 30 min and then centrifuged at 2500*g* for 10 min. One ml aliquots of the supernatant, in duplicate, were transferred to new Pyrex tubes (fraction 2, water-extractable AX). All fractions were diluted to 2 ml and 10 ml of freshly prepared extraction solution (93.2% (v/v) acetic acid (Sigma), 1.69% (v/v) hydrochloric acid (Fisher Scientific), 0.85% (w/v) phloroglucinol (Sigma) and 0.017% (w/v) glucose (Sigma) was added to each sample. Samples were boiled for 25 min and vortexed frequently. After rapid cooling the absorbance of the resulting solution was measured. The pentosan content was determined by comparing the differences in absorbance measured at 552 and 510 nm. Values were calculated based on a calibration curve generated using known amounts of xylose (Sigma) standard. AX solubility was calculated as the ratio of WE-AX to TOT-AX. Each sample was analysed with three technical replicates.

### 2.4 Enzyme fingerprinting of arabinoxylan and β-glucan

Enzyme fingerprinting of AX and mixed MLG was as described by Ordaz-Ortiz et al. (2004), Ordaz-Ortiz and Saulnier (2005). Flours were digested with endo 1,4 β-xylanase (E.C.3.2.1.8), a xylanase of the GH11 group, and endo 1,3(4) glucanase (‘lichenase’) (E.C.3.2.1.73) to digest AX and MLG, respectively from Megazyme. Briefly, 1 ml of 80% (v/v) ethanol was added to 100 mg of flour and heated in a 95 °C water bath for 10 min to inactivate endogenous enzymes present in the samples. After centrifugation (10,000*g* 5 mins RT), the residue was washed with 80% (v/v) ethanol and then with 95% (v/v) ethanol to remove free sugars, and dried using a Speedvac centrifugal evaporator. The dried powder was resuspended in 1 ml of water containing 2 µl (16 U) of endoxylanase and 1 µl (2 U) of lichenase and incubated at 40 °C for 16 h with continuous rotation in a Thermomixer (Eppendorf). Samples were then centrifuged at 13,400*g* for 5 min, the supernatants boiled for 30 min to inactivate hydrolases and filtered through 0.45 µm PVDF filter (Whatman). Finally, samples were diluted 1:20 with water prior to separation by HPAEC-PAD (Dionex ICS-3000, Thermo Scientific) equipped with a CarboPac PA1 analytical column. Two technical replicates were analysed for each biological replicate. The peak areas of the oligosaccharides released by enzyme digestion of AX (AXOS) were expressed as percentages of the total peak areas of all AXOS. The two major glucooligosaccharides (GOS) released by enzymatic digestion of MLG by lichenase comprised three glucose residues (G3) and four glucose residues (G4). MLG was therefore calculated as the sum of G3 + G4 peak areas, and G3 to G4 ratio calculated.

### 2.5 Determination of relative viscosity of aqueous extracts

Aqueous extracts were prepared from semolina as described by Saulnier et al. (1995) but with an additional centrifugation step at 10,000 *g* for 10 min at room temperature before filtration. They were stored on ice prior to measurement of relative viscosity (ηrel = t/t0, where t0: flow time of distilled water, 72–74 s) at 30 °C using an automated viscometer (AVS 370, SI Analytics, Germany) fitted with an Ostwald capillary tube (2 ml, diameter 0.4 mm). Values are the means of two extractions with the flow time of each extract being measured five times (Saulnier et al., 1995).

### 2.6 Statistical analysis

**Fig. 1:**
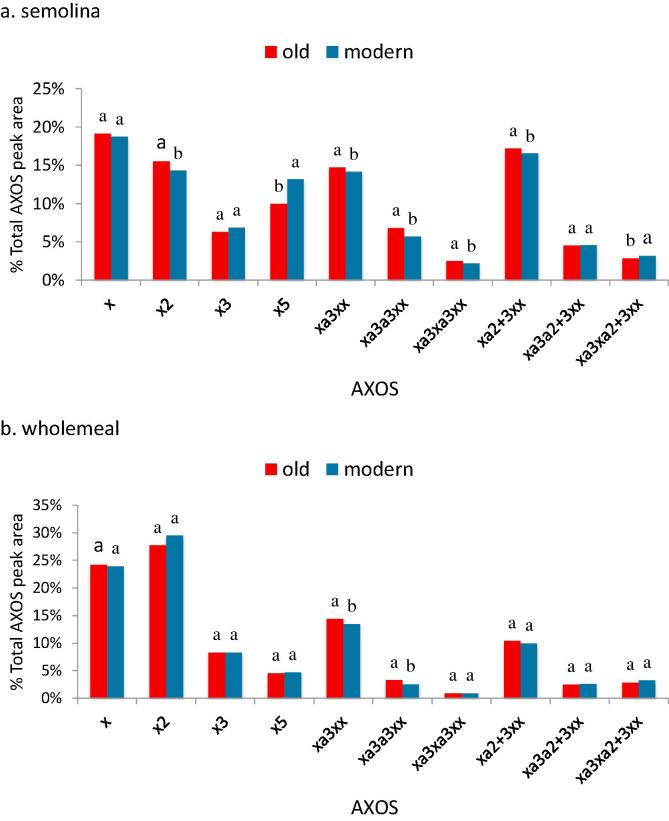
Relative proportions of AXOS released by endoxylanase digestion of AX in semolina (a) and wholemeal flour (b) of old and modern groups of durum wheat genotypes grown in two years (2013 and 2014). Values with different letters are significantly different at P ≤ 0.05 according to the Student’s *T*-test.

**Fig. 2:**
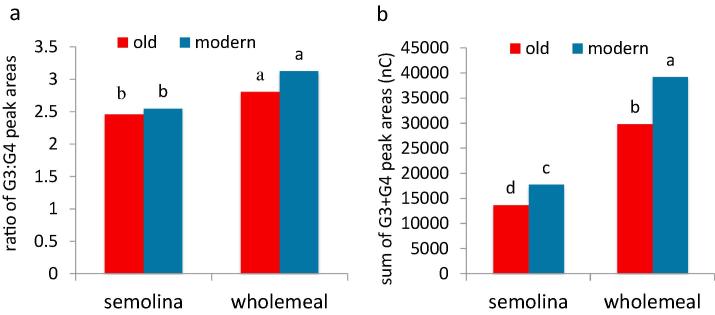
Ratio of the areas of the G3 to G4 GOS (a) and the sum of G3 and G3 GOS peak area (b) released by lichenase digestion MLG in semolina and wholemeal flours of old and modern groups of durum wheat genotypes grown in two years (2013 and 2014). Values with different letters, respectively within semolina and wholemeal flour, are significantly different at P ≤ 0.05 according to the Student’s *T*-test.

Two-way analysis of variance (ANOVA) was carried out using as factors genotype and crop season (Table1a). Tukey’s test was used post hoc. The means of the two groups were compared by Student’s *T*-Test (Table1b, Fig. 1, Fig. 2). Principal component analysis (PCA) was performed separately on the correlation matrices comprising DF parameters of the semolina and wholemeal flours. The values of each variable were standardised before performing PCA and the Varimax method used to give the best orthogonal factor rotation. The PCA results were graphically represented in two-dimensional plots. Statistical analysis was performed with software JMP (Version 8.0.2, SAS Institute Inc., 2009).

## 3 Results

### 3.1 General

Groups of seven older (dating from 1900 to 1949) and eight modern (dating from 1985 to 2005) Italian durum wheat genotypes were grown in replicated randomised field trials for two seasons. ANOVA showed significant interactions between the two groups of genotypes (old and modern) and the crop season (Table 1 in De Santis et al., submitted). Thousand kernel weight (TKW) and test weight (TW) reflect the size and shape of the kernels. There was no significant difference between the mean TKWs of the old and modern groups of genotypes in 2013, but the modern genotypes had a significantly lower mean TWK than the old genotypes in 2014. Similarly, the mean TW did not differ significantly in 2013 but was significantly lower in the old group of cultivars in 2014 while the grain protein content (GPC) and semolina protein content (SPC) were generally higher in the old genotypes, except for the modern cultivar Svevo, but the mean values for these parameters were only significantly different in 2013. The ash content (which reflects grain mineral content) of wholemeal was significantly higher in 2014 in the modern group of genotypes only.

### 3.2 Total and water-extractable pentosans (arabinoxylan)

The contents of TOT-AX and WE-AX in wholemeal and white flours of the lines were determined using an assay for pentosans (Table 1).

TOT-AX in semolina ranged from 1.4 to 1.8% dry weight and in wholemeal 3.3 to 4.6% dry weight (Table 1). However, whereas ANOVA showed no significant differences between TOT-AX in the semolina fractions, highly significant differences due to the genotype, crop season (Y) and G × Y interactions (F significance ≤ 0.001) were observed in wholemeal, with significantly higher values being observed for the older genotypes old Saragolla and Russello and the recent genotypes Svevo and Saragolla in 2014. However, no significant differences were observed between the mean values for TOT-AX in the groups of old and recent genotypes within crop years.

Wider variation was observed in the WE-AX contents of the samples, from 0.3 to 0.8% dry weight in semolina and from 0.4 to 1.0% dry weight in wholemeal (Table 1). In general, significantly higher contents of WE-AX were observed in semolina in 2014, in both the old and modern groups, with significantly higher contents of WE-AX in the wholemeal flours of the modern compared to the old genotypes in both crop years.

The variation in WE-AX content between crop years and genotypes resulted in differences in % AX solubility, which ranged from 18% to 46% in semolina and from 9% to 24% in wholemeal flour. The % AX solubility in semolina was generally higher in 2014 than in 2013, with no significant difference between the means for the old and modern cultivars. By contrast, the year effect was less clear in wholemeal, but the mean % AX solubility was significantly higher in the modern than in the old group of genotypes.

### 3.3 Relative viscosity

The relative viscosity (RV) of aqueous extracts (compared to water) of wheat flour/semolina was determined by the concentration of AX and, to a lesser extent MLG, and significant correlations were found between RV, WE-AX content and AX solubility (Table 4 in De Santis et al., submitted). Wide variation in RV was observed (Table 1), with values ranging from 1.29 to 2.30 and significantly higher values for both groups of genotypes in 2014. The relative viscosity was also significantly higher for the modern than the older genotypes in 2014.

### 3.4 Arabinoxylan structure

The structure of AX in the wholemeal and semolina flours was compared by determining the proportions of oligosaccharides (AXOS) released by digestion with endoxylanase (enzyme fingerprinting). The most abundant oligosaccharides released from both flours were xylose (x), xylobiose (x_2_), and the monosubstituted AXOS xa^3^xx and/or the disubstituted AXOS xa^2+3^xx. The proportions of x_,_ x_2_ and x_3_ were higher in the wholemeal fractions with higher proportions of substituted AXOS in the semolina fractions.

Statistically significant differences in the proportions of individual AXOS were observed between genotypes and years (Tables 2 and 3 in De Santis et al., submitted). However, in most cases these differences were small, accounting in most cases for less than 2% of the total AXOS peak area, with the overall patterns being remarkably similar. The only difference greater than 2% in the means for the two years was for the x_5_ AXOS, with means of 10% and 13.20% total AXOS peaks in semolina of the old and recent groups of genotypes, respectively (Fig. 1). These results therefore demonstrate that modern breeding has had little impact on AX structure in semolina or wholemeal of Italian durum wheats.

### 3.5 Amount and structure of β- glucan

Digestion of MLG with lichenase releases two major glucooligosaccharides (GOS) comprising three (G3) and four (G4) glucose units. The sum of these fragments therefore provides a good estimate of TOT-MLG while the ratio of G3:G4 GOS provides information on the structure (relative numbers and distributions of β1-3 and β1-4 bonds) (Fig. 2).

Clear differences were observed between the contents of G3 + G4 fragments in the semolina and wholemeal flours with ANOVA showing a significant effect of the G × Y interaction. In particular, the mean total contents of G3 + G4 GOS were significantly higher in the semolina and wholemeal flours of the modern compared with the older group of genotypes in both crop years (Fig. 2), while the total contents in wholemeals of both groups of varieties were higher in 2014 than in 2013 (Tables 3 and 4 in De Santis et al., submitted).

The ratio of G3 and G4 GOS (Fig.2a) ranged widely, from 1.98 (Iride, 2013) to 3.16 (Preco, 2014) in semolina and from 2.27 (Grifoni 235, 2013) to 5.71 (Adamello, 2014) in wholemeal, but the means for semolina and wholemeal were significantly higher in 2014 than in 2013, and for the modern lines in wholemeal in 2014 only (Tables 3 and 4 in De Santis et al., submitted).

### 3.6 Principal component analysis

The analyses reported above clearly show that there were limited, but significant, differences between the compositions of the genotypes and between crop years. To obtain a clearer picture of the variation in AX, MLG and RV of aqueous extracts the datasets were subjected to principal component analysis (PCA) on the correlation matrix (Table 5 in De Santis et al., submitted).

**Fig. 3:**
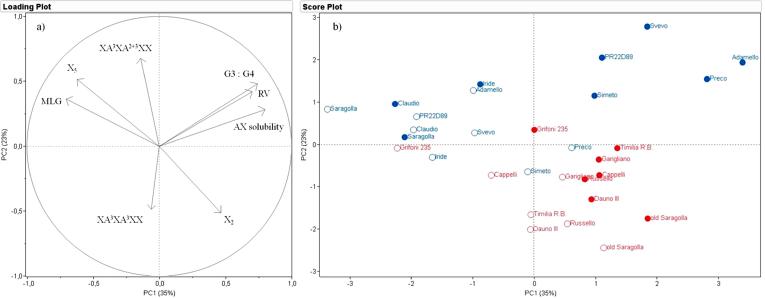
PCA of the content and composition of dietary fibre components (AX and MLG) and the relative viscosity of aqueous extracts of semolina flour. a, loadings plot showing the contributions of parameters to the separation; b, distribution of samples of old (red) and modern (blue) genotypes grown in 2013 (empty symbols) and 2014 (solid symbols). (For interpretation of the references to colour in this figure legend, the reader is referred to the web version of this article.)

Analysis of the data for semolina showed that PC1 and PC2 explained 35% and 23% of the observed variation, respectively (Fig.3a) with the loadings plot (Fig.3b) showing that the separation in PC1 was mainly determined by AX solubility, RV and the amount and structure of MLG (Fig.3a, loadings plot), while PC2 was mainly affected by AX structure determined by fingerprinting. Although the old genotypes are concentrated in the lower part of the plot and the recent genotypes in the upper, with the 2013 samples to the left and the 2014 samples to the right, some overlapping of the sample sets was also observed.

**Fig. 4:**
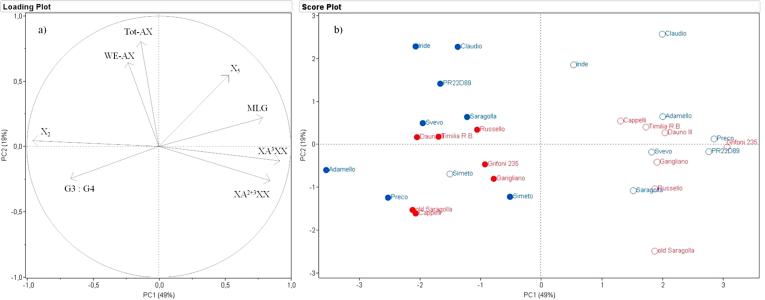
PCA of the content and composition of dietary fibre components (AX and MLG) in wholemeal flour. a, loadings plot showing the contributions of parameters to the separation; b, distribution of samples of old (red) and modern (blue) genotypes grown in 2013 (empty symbols) and 2014 (solid symbols). (For interpretation of the references to colour in this figure legend, the reader is referred to the web version of this article.)

Analysis of the dataset for wholemeal flours (Fig.4a) showed that PC1 and PC2 accounted for 49% and 19% of the observed variability, respectively, with the loadings plot (Fig.4b) showing that the separation in PC1 was determined by MLG content and structure and AX structure determined by fingerprinting, and PC2 mainly by TOT-AX content but with a contribution from MLG structure and content. Although the 2013 and 2014 samples were clearly separated in PC1, on left and right hand sides of the plot, respectively, no clear separation between the old and recent groups of genotypes was observed.

## 4 Discussion

### 4.1 Content and composition of AX and MLG in wholemeal and semolina

The contents of TOT-AX and WE-AX determined in this study are within the range reported for durum wheat (Ciccoritti et al., 2011, Gebruers et al., 2008, Marcotuli et al., 2015), as are the values for relative viscosity (Ordaz-Ortiz, Devaux, & Saulnier, 2005). Ordaz-Ortiz et al. (2005) also reported similar results for enzyme fingerprinting of five milling fractions from two French bread wheat cultivars, with AX fragments corresponding to xylose (x), xylobiose (x_2_) and the mono-substituted AXOS xa^3^xx being the most abundant, and x and x^2^ being predominant in wholemeal flour. The higher G3:G4 ratio determined in wholemeal flour is also consistent with the higher ratio of DP3:DP4 in bran compared with white flour (Marcotuli et al., 2016, Marcotuli et al., 2016).

The relative viscosity of aqueous extracts of semolina was related to the proportion of soluble AX. This is to be expected as WE-AX is considered to be the main determinant of this parameter. (Martinant et al., 1999). However, RV was also related to the G3:G4 ratio of MLG, which is consistent with the suggestion that proportion of the G3 GOS plays a role in determining MLG solubility and extract viscosity (Lafiandra et al., 2014).

### 4.2 Effect of breeding on DF composition

The 15 durum wheat genotypes analysed ranged from types grown at the beginning of the 20 th century (old Italian landraces) to modern cultivars released up to 2005. Significantly higher contents of WE-AX were observed in the wholemeal flours of the modern compared to the old genotypes in both crop years, but no differences in the contents of WE-AX in semolina, or in TOT-AX in either flour. Nevertheless, the recent cultivars showed significantly higher mean values for % AX solubility in both semolina and wholemeal. Similarly, no relationships were found between the contents of AX (TOT and WE) in flour and bran of 150 bread wheat lines in the HEALTHGRAIN diversity screen and their release dates (Shewry et al., 2011), or in the contents of TOT-AX in whole grain of either two old and seven modern durum genotypes (Marotti et al., 2011) or three groups of durum wheat genotypes with different release dates (Marcotuli et al., 2015).

Enzyme fingerprinting showed some differences in the structure of AX between the old and modern genotypes, which were greater in extent in semolina than in white flour. However, we are not aware of any other study on the relationship between AX structure and release date as the most extensive published study of variation in AX structure (of the 150 HEALTHGRAIN bread wheat lines (Shewry, Saulnier, et al., 2010)) did not analyse the patterns of AXOS in this respect.

By contrast to AX, the mean contents of MLG, calculated as the sum of the G3 and G4 peak areas from enzyme fingerprinting, were about 30% higher in the group of modern genotypes, both in semolina and in wholemeal. This contrasts with the HEALTHGRAIN study which showed no relationship between the content of MLG in wholemeal and the year of release (Shewry et al., 2011), although it should be noted that this study was carried out on single replicate samples of bread wheat genotypes grown in a single year.

### 4.3 Effect of the crop season on DF composition

Although no significant differences were observed between the mean values for TOT-AX in wholemeal or semolina, significantly higher contents of WE-AX were observed in semolina in 2014 and these were associated with higher values for RV of aqueous extracts. These differences may have related to the higher rainfall during grain development in 2014 (De Santis et al., 2017) as Shewry, Piironen, et al. (2010) reported positive correlations between rainfall from heading to harvest and the contents of WE-AX in flour (0.692) and bran (0.737) of modern bread wheat cultivars. Ciccoritti et al. (2011) similarly showed effects of environment and of genotype × environment interactions on AX in 19 durum wheat cultivars grown in four environments while Li, Morris, and Bettge (2009) reported effects of environment on AX in sets of 25 spring and 25 winter bread wheat cultivars grown in 3 environments each. However, neither study was able to ascribe the effects to specific environmental factors. The total contents of MLG in wholemeal were also higher in 2014 than in 2013 and the mean G3:G4 ratios higher in both semolina and wholemeal. Although Shewry, Piironen, et al. (2010), reported that the total amount of MLG in wholemeal of bread wheat was negatively correlated with the precipitation between heading and harvest, this relationship was not statistically significant (*p* = .134). Finally, the older genotypes tended to show greater stability in content and composition of AX and MLG than the modern genotypes, although exceptions to this were observed (notably old Saragolla).

## 5 Conclusions

Comparisons of the contents and structures of AX and MLG in groups of old and modern durum wheat genotypes has provided no evidence that intensive breeding has had negative effects on the contents of dietary fibre components in durum wheat. In fact, the modern genotypes had higher contents of WE-AX in wholemeal and higher mean values for % AX solubility in both semolina and wholemeal than the older genotypes. The mean contents of MLG were also about 30% higher in semolina and wholemeal of the group of modern genotypes. The relative viscosity of aqueous extracts of semolina was also significantly higher for the modern than the older group of genotypes in 2014 only. The identification of modern cultivars with high viscosity associated with a high content of MLG suggests that they are good sources of dietary fibre for human health.
