# Supplementary material for: Comparison of the dietary fibre composition of old and modern durum wheat (Triticum turgidum spp. durum) genotypes
Source: Food Chem. 2018 Apr 1;244:304–10. doi: 10.1016/j.foodchem.2017.09.143 (PMC5692191; doi:10.1016/j.foodchem.2017.09.143)
Supplement: Supplementary data — Supplementary Fig. S1. [file mmc1.docx]

**Supplementary data**


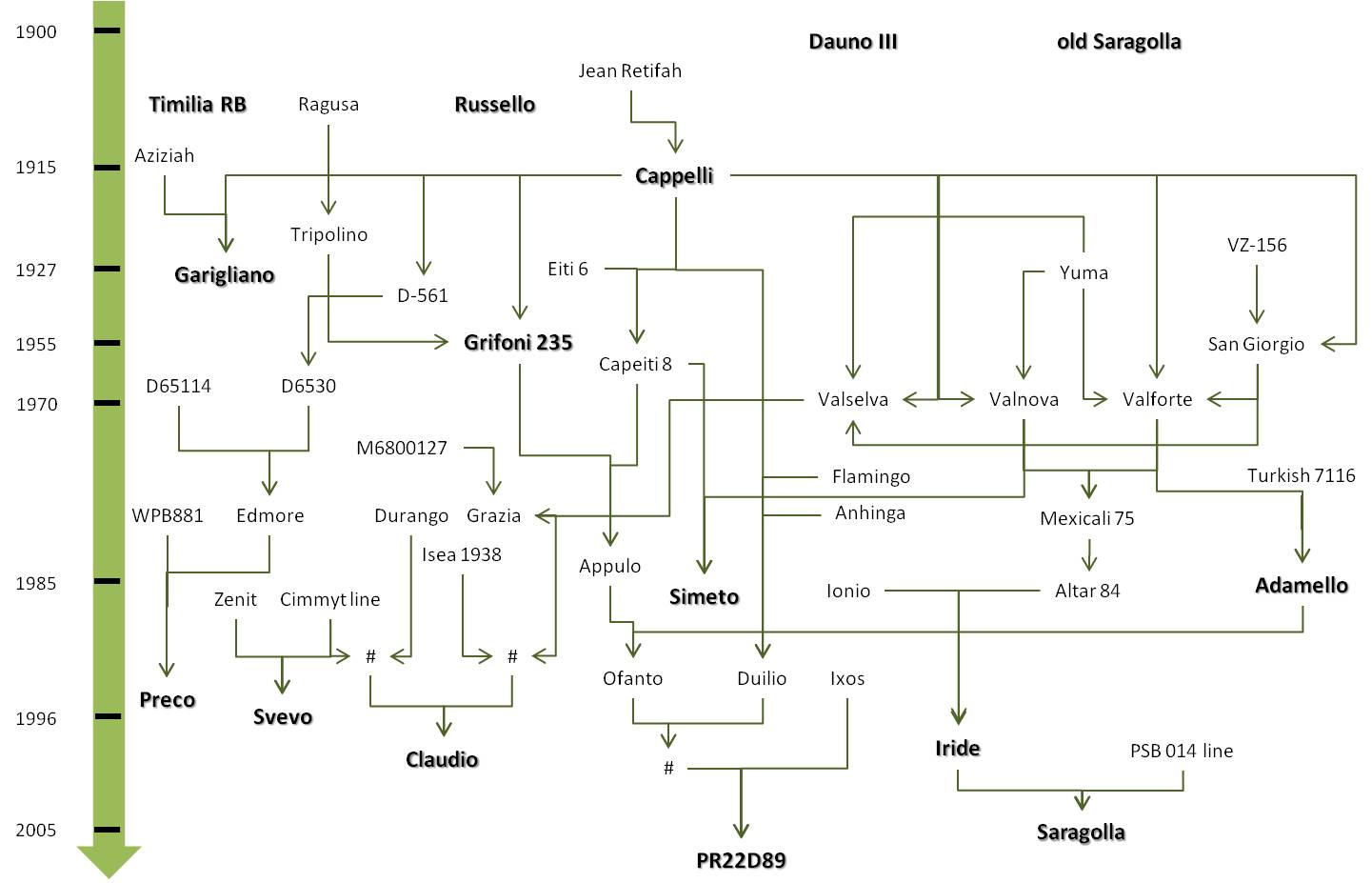


**Figure S1**. Genetic pedigree of Italian durum wheat genotypes investigated for dietary fibre composition
